# Supplementary material for: A Qualitative Photo Elicitation Research Study to elicit the perception of young children with Developmental Disabilities such as ADHD and/or DCD and/or ASD on their participation
Source: PLoS One. 2020 Mar 18;15(3):e0229538. doi: 10.1371/journal.pone.0229538 (PMC7080235; doi:10.1371/journal.pone.0229538)
Supplement: S1 Appendix — (DOCX) [file pone.0229538.s001.docx]

**Appendix 1: Interview protocol**

Composed following the guidelines and advice of Whyte (2005) and Curtin (2001).

**First visit: Getting to know you**

- Greet the child (and the parent) in its preferred way.
- Provide a drink for the child and parent.
- Explain the research project, visits, camera assignment and informed consent.
- Provide written informed consent for the parent to follow and sign.
- Explain to the child in an age-appropriate language use.
- While the parent overhears.
- Use props to help explain.
- Encourage the child and parent to ask questions. Ask the child to articulate its understanding of the research.
- Ask the child where everybody should sit and if it wants to help set up the camera (overcoming inequality).
- Start the preferred activity
  - Ask the child (if it wants) to talk about the picture or drawing (overcoming inequality: giving a position of expertise).
- Interview questions: Let the child guide the conversation. When the child stops talking, ask open questions to encourage it to elaborate on its experiences. Let the child select what question (visual aid) to answer (overcoming inequality).
  - Tell the child that every answer is a good answer. You know absolutely nothing and really want to hear what the child thinks (examining beliefs & overcoming inequality) but even so it’s okay if it doesn’t know or want to give an answer.
  - What do you like to do?
  - What did you do today/yesterday/this week?
  - Where? When? How often? For how long? With whom?
  - How does it make you feel?
  - What do you think about that?
  - Tell me more.
  - Paraphrasing the last sentences.
- When the child does not understand or answer the question, first examine your part in this occurrence. For example: asking a vague question (bridging different styles of communication, examining beliefs: maintaining a non-adult-centric view).
- Games or activities for when the child is shy or demotivated can be used.
- Give the possibility of choosing their own pseudonym.
- Ask the child to repeat the camera assignment and give the camera.
- Ask the child and parent for comments on the interview.

**Camera assignment: You’re the photographer**

- The child borrows the camera for one week, wears it around its neck or waist and tries to take at least one picture of everything it does.
- Provide an accompanying letter for family members, teachers, leisure supervisor, …

**Second visit (week after first visit): Picture time**

- Bring a snack for the child and parent.
- Ask the child where everybody should sit and if it wants to help set up the camera (overcoming inequality).
- Discuss with the child the analysis of previous session. Let them choose their own pseudonym.
- Tell the child that every answer is a good answer. You know absolutely nothing and really want to hear what the child thinks. But even so it’s okay if it doesn’t know or want to give an answer.
- Tell the child it’s okay to notify you when it is tired or bored. If so, have a short break.
- Discuss the pictures taken for the camera assignment. Ask the child what picture to discuss first/next (overcoming inequality).
  - Encourage elaboration via open questions (using the visualizations).
  - Tell me more about this picture.
  - Where/when/who is this?
  - What were you feeling/thinking at that moment?
  - Paraphrasing the last sentences.
- Ask the child and parent for comments on the interview.

**Third visit (preferably one week after second): Thank you and goodbye for now**

- Ask the child where everybody should sit and if it wants to help set up the camera (overcoming inequality).
- Tell the child it’s okay to notify you when it is tired or bored. If so, have a short break.
- Tell the child that every answer is a good answer. You know absolutely nothing and really want to hear what the child thinks. But even so it’s okay if it doesn’t know or want to give an answer. Cover topics left over from last session.
- Present the child with the data analysis and -synthesis of its interviews. Check if the formulated interpretations are correct.
- Are you happy with what you do/can do?
- What do you want to do more/less/different?
- Can you do everything you want to do?
- Ask for ideas on how to disseminate the findings of the study.
- Ask the child and parent for comments on the interview.
- The child picks a thank you gift.

**Attitude and points of consideration for the researcher**

- Examine your own beliefs
  - Maintain a non-adult-centric view. Recognize children as experts about their own lives.
- Overcome inequality
  - Refrain from behavior that could be interpreted as authoritarian, judgmental or interfering. Don’t use judgmental phrases like “that’s right” or “that’s good”.
  - Maintain a responsive versus a dominating stance towards the child.
- Bridge communication styles:
  - Learn the vocabulary and phrase length the child uses. Ask questions that are a maximum of 5 words longer than the child’s average numbers of words in a sentence.
  - Refrain from using complex adult language.
  - Keep in mind to use non-verbal conversation techniques.
  - Ask questions about the immediate/observable situation.
  - Develop questions that use words introduced by the child.
  - Use names instead of pronouns.
  - Avoid “why”-questions.
  - Ask the child to repeat the question to check if they understood it.

**References**

Curtin, C. (2001). Eliciting children’s voices in qualitative research. *American Journal of Occupational Therapy, 55*(3), 295-302.

Whyte, J. (2005). Research with children with disabilities: Guidelines and checklist for good practice: Children's Research Centre, Trinity College Dublin.
