# Supplementary material for: A Qualitative Photo Elicitation Research Study to elicit the perception of young children with Developmental Disabilities such as ADHD and/or DCD and/or ASD on their participation
Source: PLoS One. 2020 Mar 18;15(3):e0229538. doi: 10.1371/journal.pone.0229538 (PMC7080235; doi:10.1371/journal.pone.0229538)
Supplement: S2 Appendix — (PDF) [file pone.0229538.s002.pdf]

Hey! Ik ben Juul.

Ik doe heel veel dingen. Schoolgaan. Spelen. Helpen thuis. En nog veel meer. Vaak kan ik doen wat ik wil. Soms ook niet.

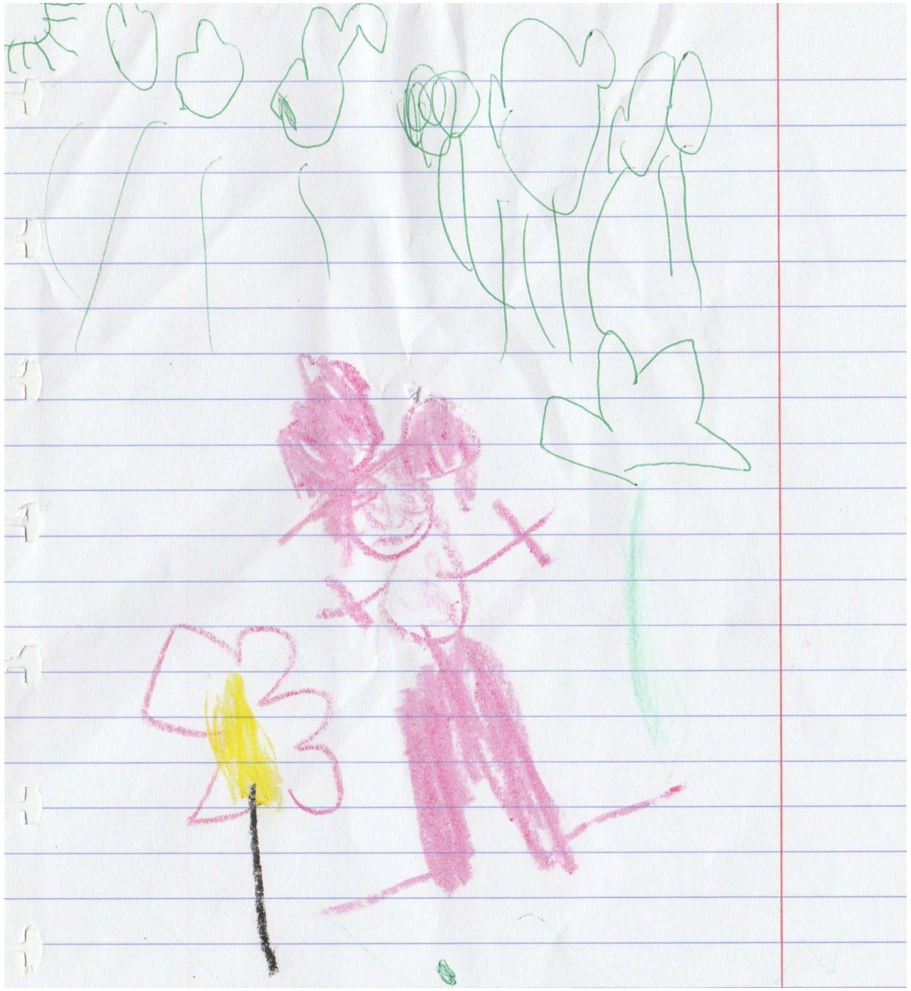

Soms kan ik kiezen wat ik doe. Soms is het mooi weer als ik sport. Soms helpt iemand mij. Soms zijn er veel kindjes om samen te werken. Soms betaalt mama een ijsje. Dat vind ik allemaal leuk.	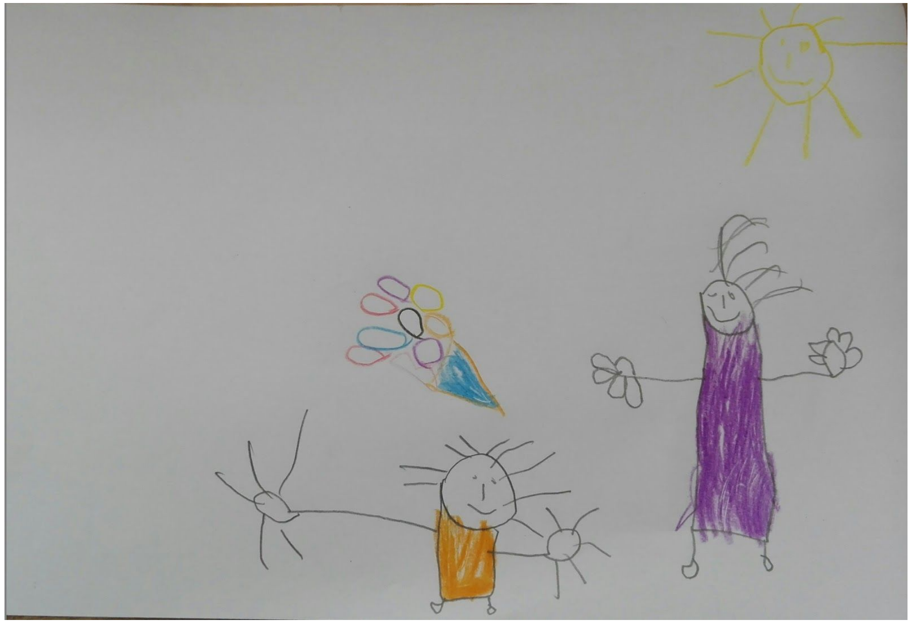

Soms mag ik niet meespelen. Soms kan papa me niet voeren. Soms is het spel te moeilijk. Soms is het saai. Dat vind ik niet leuk.

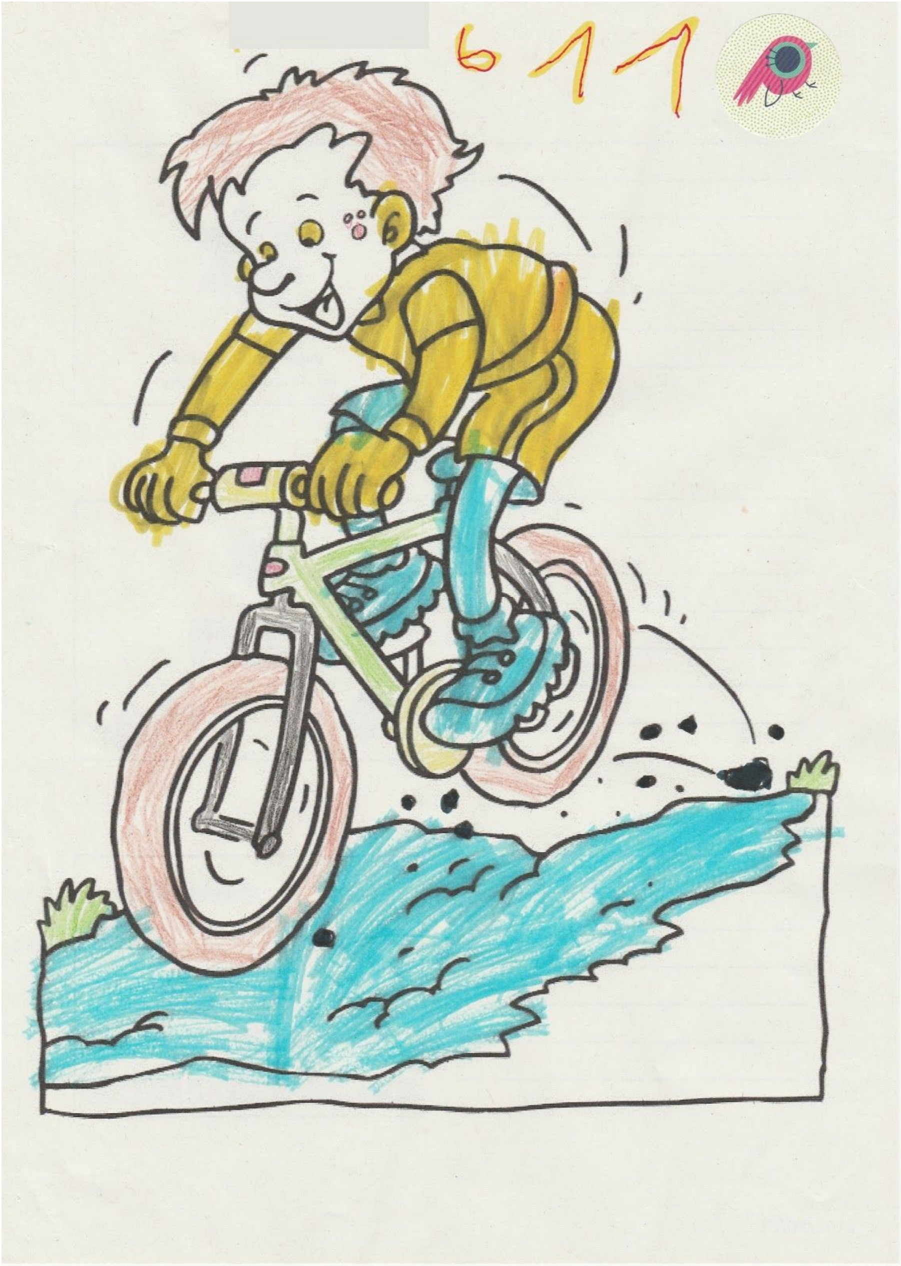

Ik heb dit allemaal verteld aan onderzoekers. Andere kindjes ook. Wij hebben de onderzoekers slimmer gemaakt. Zij kunnen nu kindjes helpen om meer leuke dingen te doen.	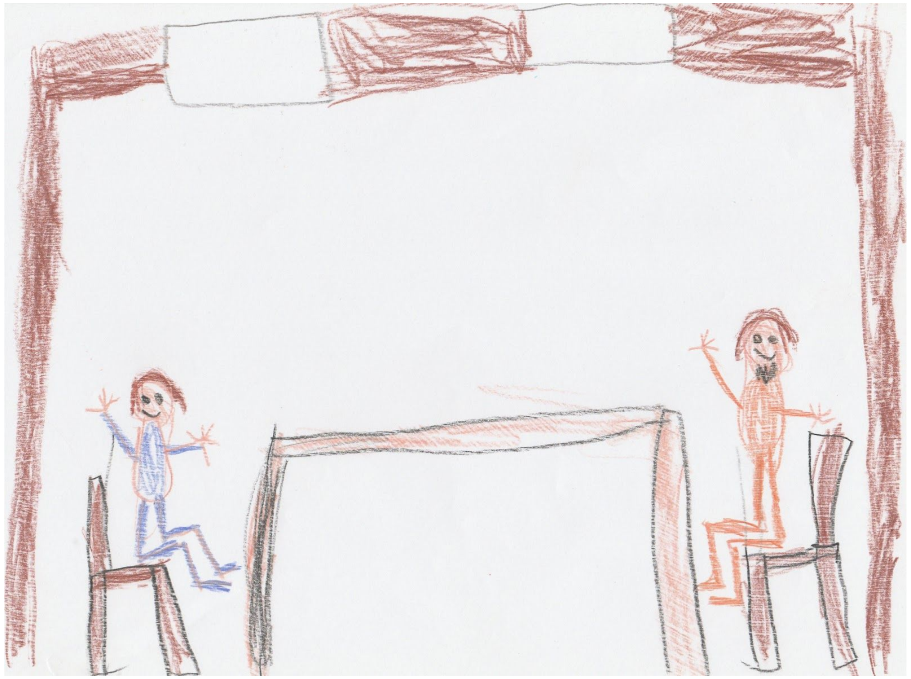
