# Supplementary material for: A Qualitative Photo Elicitation Research Study to elicit the perception of young children with Developmental Disabilities such as ADHD and/or DCD and/or ASD on their participation
Source: PLoS One. 2020 Mar 18;15(3):e0229538. doi: 10.1371/journal.pone.0229538 (PMC7080235; doi:10.1371/journal.pone.0229538)
Supplement: S3 Appendix — (DOCX) [file pone.0229538.s003.docx]

**Appendix 3: Short summary of the Photographs**

**Pictures taken at home**

| **Bed- and meal time routines** | **Family members** | **Family gatherings** | **Playing** | **Pets** | **Household chores** |
| --- | --- | --- | --- | --- | --- |
| Going to bed with the cat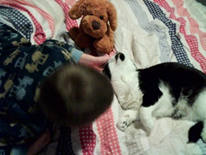 | Mother = secret weapon  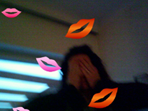 | Celebrations  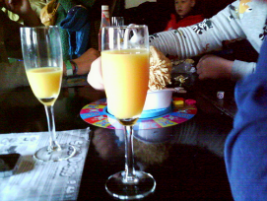 | Playing with Lego  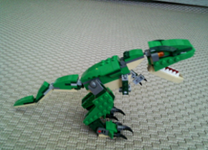 | The family cat  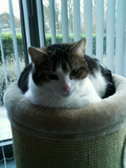 | Helping mom with the dishes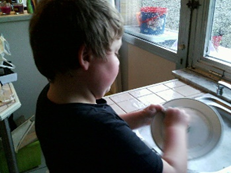 |
| In bed with stuffed animals  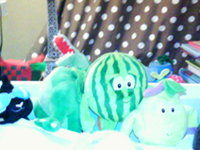 | Grandparents  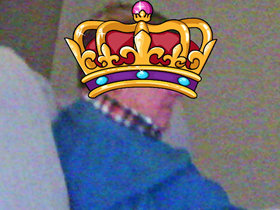 | Family trips  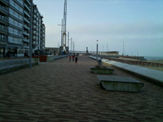 | Playing with Dino’s  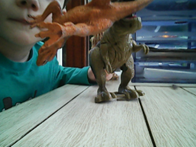 | The family dog  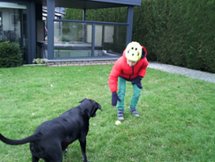 | Helping parents to light up the fire  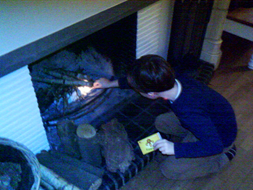 |
| Enjoying family meals  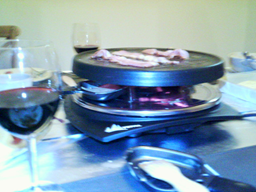 | Siblings  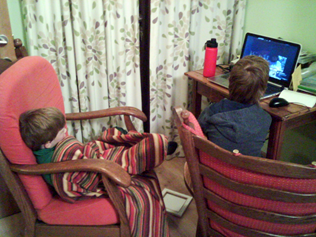 | Going to the zoo by train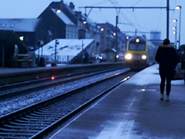 | Playing with cars  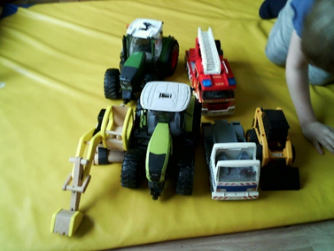 | The fish  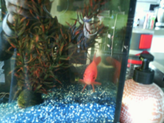 | Helping mom with cleaning  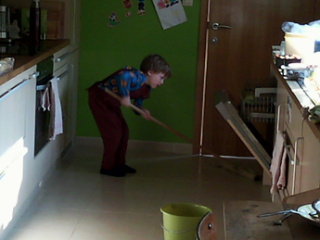 |
| Enjoying their bedtime routine with mom  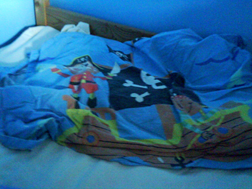 | Having fun with Dad  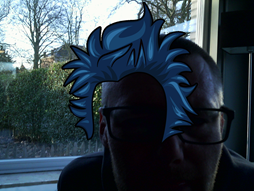 | Watching TV  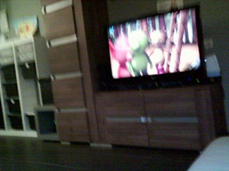 | Playing with siblings  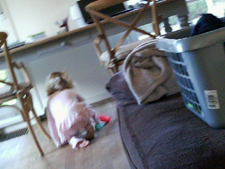 | The chickens  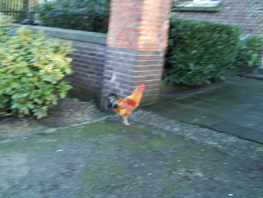 | Going shopping  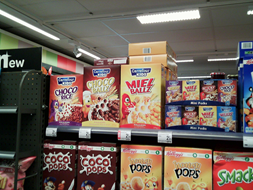 |

**Pictures taken in the community**

| Going to a sport club  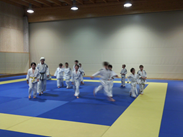 | Playing in playgrounds  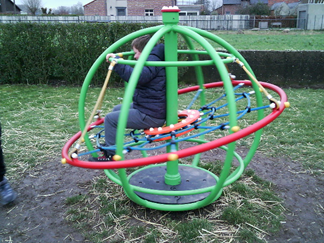 | Learning to bike  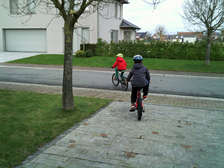 | Using the step  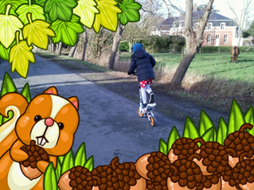 |
| --- | --- | --- | --- |

**Pictures taken at school**

| **During Class** | **During Recess** | **During school trips** |
| --- | --- | --- |
| Doing sport at school  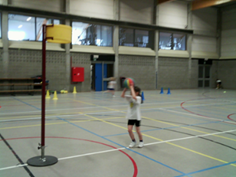 | Playing with friends  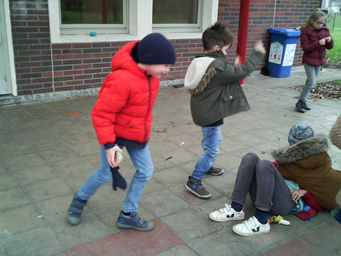 | Visiting nearby monuments  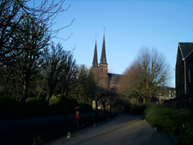 |
| Doing arts project  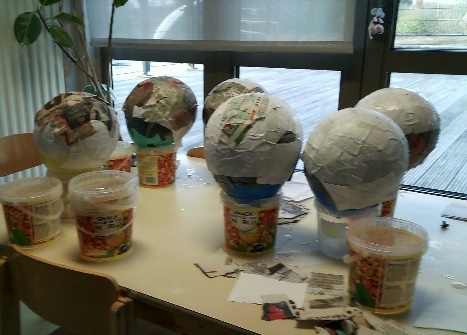 | Playing in the sandbox  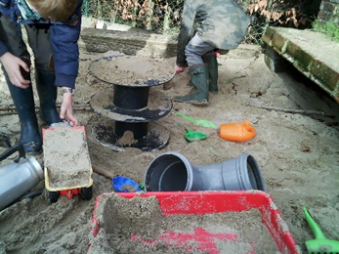 | Visiting the library  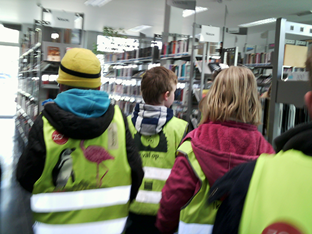 |
| Learning math and language  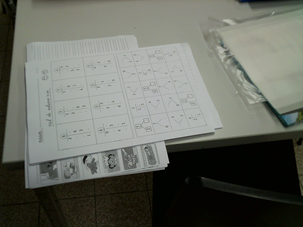 | Enjoying the nature  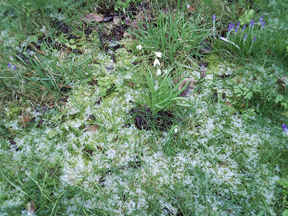 | Going to the forest  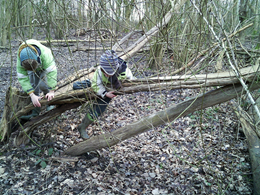 |
