# Supplementary material for: A Qualitative Photo Elicitation Research Study to elicit the perception of young children with Developmental Disabilities such as ADHD and/or DCD and/or ASD on their participation
Source: PLoS One. 2020 Mar 18;15(3):e0229538. doi: 10.1371/journal.pone.0229538 (PMC7080235; doi:10.1371/journal.pone.0229538)
Supplement: Attachment — Submitted filename: Response to revierwers.docx [file pone.0229538.s004.docx]

**Point by point answer to the reviewers.**

Dear Editor,

Dear reviewers,

Thank you for this additional feedback.

We have tried to make all necessary changes and a point to point response can be found below.

We hope this responds to the remarks, but we are willing to make any changes if this is not the case.

Sincerely,

Marieke Coussens, on behalf of the entire research team.

| **Comments from the Editor** | **Response** | **Changes in the manuscript** |
| --- | --- | --- |
| We noticed you have some minor occurrence of overlapping text with the following previous publication(s), which needs to be addressed | Indeed the suggested literature are known to the research team. Thanks for pointing this out. We made changes in the manuscript (in blue, bold and italic to make it visually easier to trace the changes made in the manuscript). | The changes in the manuscript are written in bleu, **bold** and *Italic.* We also highlighted in yellow in which section of the manuscript we made the changes and we added the numbers of the lines. We also followed the track changes system. We hope that we – by doing so- made it easier for you to follow. Al the number lines are based on the document ‘clean manuscript’. |
| Maciver, Donald, et al. "Participation of children with disabilities in school: A realist systematic review of psychosocial and environmental factors." PloS one 14.1 (2019): e0210511. | The reference you suggest (Maciver, 2019) has been already been included in the original manuscript and we added it extra as well in the introduction section. | In the introduction section, we additionally added the reference on line 64: “Despite this emphasis on participation (Dijkers 2010, Imms 2017, Van de Velde 2017, ***Maciver, Rutherford, Arakeylyan, Kramer, Richmond, Todorova et al., 2019)*** there seems to be no consensuses on how participation should be interpreted.  In the introduction section, on line 73, we already had the reference of Maciver included. I highlighted it this time to make it more clear. Both Imms et al. and Maciver et al. (Imms 2017, ***Maciver 2019***) highlighted the importance of a careful definition of participation |
| Antshel, Kevin M., and Natalie Russo. "Autism spectrum disorders and ADHD: Overlapping phenomenology, diagnostic issues, and treatment considerations." Current psychiatry reports 21.5 (2019): 34. | That has been a mistake on my account. Our apologies for this. We made changes in the manuscript. | In the introduction section, on line 98 we included the suggested reference. ADHD, DCD and ASD are neurodevelopmental disorders which typically onset in early childhood (***Antshel & Russo, 2019***). |
| Zwicker, Jill G., et al. "Developmental coordination disorder is more than a motor problem: children describe the impact of daily struggles on their quality of life." British journal of occupational therapy 81.2 (2018): 65-73. | We added those authors in the manuscript. | In the introduction section on line 128 we added the suggested reference.  In addition, children with DCD were found to experience difficulties with dressing up, tying shoes, catching a ball, physical education, play skills and engagement in leisure activities (Polatajko 2005, Missiuna 2007; ***Zwicker, Suto, Harris, Clasakova & Missiuna,2018)*** due to their motor coordination problems, resulting in low participation in socially valued leisure activities, such as team sports. |
| The text that needs to be addressed involves some sentences of the introduction. | Thank you for pointing this out. We have made the necessary changes in the manuscript. |  |
| In your revision ensure you cite all your sources (including your own works), and quote or rephrase any duplicated text outside the methods section. | Thank you for this remark; we have cited all the sources. Also, you suggested to add own work. We already did so in the original manuscript. We also payed careful attention that if we did use a quote or rephrased/duplicated text in the other sections, that it was properly referenced. | In the introduction section, in the original manuscript, on line 64 the following reference was already added. I am one of the co-authors of that article. On line 67: Despite this emphasis on participation (Dijkers 2010, Imms 2017, ***Van de Velde 2017)*** there seems to be no consensuses on how participation should be interpreted. Therefore, we made no changes regarding this remark in the revised manuscript. |
| Further consideration is dependent on these concerns being addressed. | I hope the changes made in the manuscript are sufficient and that further consideration of publishing this article based on those three concerns is sufficiently addressed. | No changes |
| Please include a caption for figure 2 | Thank you for this comment. We have changed this in the manuscript. | In the result section on line 563 the following change has been made:  *Figure* ***2*** *about here* |
| Although the Reviewers appreciated the Authors' effort to fill a gap in qualitative research related to children with Developmental Disabilities, they have nevertheless highlighted important limitations of the study that require a careful and thorough revision, especially in its methodological aspects. I therefore recommend to the Authors to address all the Reviewers’ comments to make the manuscript suitable for publication. | Thank you for providing this feedback. It is nice to read that the reviewers appreciated the author’s effort to fill a gap in the qualitative research related to children with DD.  We did our best to comment on all the remarks and to make changes to ameliorate the manuscript. The reviewer’s comments are addressed below. |  |
| **Comments from 1°reviewer** |  |  |
| This is a timely and interesting paper that aims to address a lack of qualitative data on the experiences and perceptions of young children on their participation. However, there are a number of areas that warrant more attention to strengthen the report and augment the reporting of the study. | Thank you for the effort you took to provide feedback so that the paper would be strengthened.  We addressed the areas that needed more attention. We hope that this augment the chances of publication of our manuscript. | No changes |
| Research design: the research design is described as an action research study which is confusing to the reviewer as it was not defined clearly and there are many diverse ways to consider action research.  Usually however, it involves an equal attention to taking part in making some change or doing some advocacy as part of the design which is not the case in this study.  This study includes the use of photographs which can be considered participatory for sure so perhaps this is where the confusion lies.  Clarification on the description of the design and choice of methods would be important. | We indeed did not elaborate on how we defined action research. Thank you for pointing this out. We added this in the manuscript to enhance clarity. It is indeed confusing to use the term photo action research. We actually used the photographs to elicit the interview. Because our participants are young of age, we could not do the actual action nor advocacy which is inherent for this research methodology. Therefore, it might be better to describe our study as a **photo elicitating methodology** as this is what we actually did.  To meet this remark, we suggest to change the term photovoice into “photo elicitation” as the later conveys that photography was used as a reference point to elicit interview data but does not specify the source of the photos.  We therefore changed the term photo voice study into photo elicitation study throughout the entire article (title, abstract, manuscript). We hope that by doing so, we resolved this unclarity. | Changes have been made in the manuscript on different places throughout the manuscript.  In the title on line 3 we changed the manuscript title: “A Qualitative Photo-voice Action Research Study to elicit the perception of young children with Developmental Disabilities such as ADHD and/or DCD and/or ASD on their participation” into the following: ***“A Qualitative Photo Elicitation Study to evoke the perception of young children with Developmental Disabilities such as ADHD and/or DCD and/or ASD on their participation”***  In the abstract on line 27/28 we changed: “A Photo-Voice Action study was used, in which photographs were taken by the children when they were involved in meaningful activities“ into the following ***“A Photo Elicitation Study was used, in which photographs were taken by the children when they were involved in meaningful activities****”*  In the methods section on line 148 we included the following paragraph:  ***Interviewing children can be challenging for many different reasons. Difficulties in finding words, children’s stranger anxiety, different use of vocabulary, lack of spontaneous conversations and distractions due to short attention span are a few of them (Irwin & Johnson, 2005; Kirk, 2007). Hence, when interviewing children, different data collection strategies should be used to overcome these challenges. For this study we specifically choose a Photo elicitation approach. By giving children a digital camera and by letting them collect the data, we provided prompts to assist them recall events and the use of props for non-verbal demonstration of their thoughts and events during the interview. Thus, photographs were used as a tool to elicit data, not to analyze it nor to use the photographs’ as a vehicle through which findings could be represented and com­municated (Mathison, 2008) such as is expected in Participation Action Research (PAR).***  In the methods section on Line 165 we changed the term photo voice into photo elicitation methodology: Therefore, data were collected using a ***Photo elicitation study*** and has been described already as an effective method for studying the lived experiences of children with disabilities, particularly those with communication impairments (Eisen 2019).  In the methods section on Line 168 we added: The children received a digital camera and were asked to take photos ***or make movies*** of activities they did at home, in school and in their community during a week.  In the methods section on Line 171 we added the following sentence: ***More expressively efficient than mere words, the photos taken by the children were used to ease communication during the interviews (Budig et al., 2018).***  In the methods section on line 179 we added the following sentence:  ***Although the images are central, text (e.g. interview) remained the unit of analysis.***  In the discussion section on line 569 we deleted the term photo voice study and changed it into photo elicitation study. The principal goal of this ***Photo elicitation study*** was to investigate how children with DD define their participation. |
| Inclusion and exclusion criteria: in page 8, the inclusion criteria lists the diagnostic requirements of each child needing to have ' at least one of the 3 diagnoses,' then further down on line 170 it states: 'children were excluded when there was only a presumption of one of the 3 diagnoses'. Yet on page 13, line 268 there is a sentence that states: 'eight children had a comorbid disorder. This appears to imply that the remaining 8 had only 1 of the disorders, but this was an exclusion condition. Please can you clarify across the paper for consistency, what exactly the inclusion and exclusion criteria was, and to clarify consequently the list of 16 children and their profiles on table 2. | Our apology for the inconsistency regarding the in- and exclusion criteria. We rewrote this section and hope that this way of describing them made it more clear. | In the methods section on line 196 we deleted the following: ***The inclusion criteria for the children were:* *aged between 4 and 9 years of age, being diagnosed with at least one of the three diagnoses in a recognized diagnostic center, having a normal Intelligence Quotient (IQ) and being able to verbally communicate age accordingly (defined by the parents). Children were excluded when there was only a presumption of one of the three diagnose-groups.***  In the methods section on line 196 we added and changed it to the following:  The inclusion criteria for the children were:  ***(1)*** aged between 4 and 9 years of age,  ***(2)*** being diagnosed in a recognized diagnostic center with ***ADHD or DCD or ASD and another comorbid diagnosis (ADHD, DCD and/or ASD)***  ***(3) being diagnosed with ADHD, DCD or ASD and still in the diagnostic process of another diagnose such as ASD, ADHD and/or DCD***  ***(4)*** having a normal Intelligence Quotient (IQ) ***(defined by the recognized diagnostic center)*** and  ***(5)*** being able to verbally communicate age accordingly (defined by the parents).  We also deleted the following sentence in the methods section on line 196:  ***Children were excluded when there was only a presumption of one of the three diagnose-groups.***  In the results section on line 309 we added:  Two children had ASD, two had ADHD and four had DCD ***but were still in follow up for diagnostic purposes.*** |
| Reporting the findings:  Analysis of the photos is lacking in detail and the range and breadth of diverse activities is not documented. In other studies children taking photographs usually include varied activities that can include pets, nature, favorite games etc and are not only social activities which is what this study reports. | **Regarding the remark ‘analyses of the photos is lacking’:**  As the photographs were not the unit of analysis, but only used a means to elicit discussion we did not include photographs in the analysis.  However,  **To meet the remark that ‘Further detail of the synthesis of all the photos children took’ would strengthen the paper** we included a more robust profile of the range of activities that the children mentioned in their interview (based on the pictures taken). We hope that – by adding this Appendix 2 – the manuscript became more clear to the reader.  **Regarding the remark analysis is lacking regarding the range and breadth of diverse activities:**  For this study, we followed the premise that we would ‘listen’ to the children and that we would follow their lead regarding the topics that they wanted to convey during the interviews. We therefore were not searching for the breadth of diverse activities. We focussed on what the children mentioned as important to them. We do however agree with the fact that an overview of the pictures that were taken could strengthen this paper. Therfore, we have added in table 2, the number of pictures that every child took, how many movies he/she made and which photographs he/she chose to talk about in the interview.  We also added a few lines about the kind of pictures that the children took.  **Regarding the remark that ‘playing is not reflecting the full picture of participation:**  Indeed, the first theme listed is playing together. Playing does not reflect the full picture of participation. We only asked (see interview appendix) to our children to take pictures during one week so that the pictures could help the child to recall what he or she did last week. We believe that this approach facilitated the interview.  When watching the pictures or videos together with the child in the second interview, we specifically asked the children to talk about those things that were most important to them regarding their participation.  The reason why we did so is that a lot of the children took a lot of pictures just for the fun of taking pictures. Beyblade for instance took 171 pictures of cars as he wanted to know how many cars passed by during one minute. This approach enabled a child-centered and avoided a researcher-centered perspective.  However, we agree that by using this strategy, the themes mentioned might not capture the full picture of participation, which is often in the physical environment as well as the social. We therefore added this as a limitation to our study. | In the result section on line 323, we added three columns in table 2 that provides more information regarding the pictures and movies taken by the children. **Table 2: Socio-demographic characteristics *and photograph information* of the participants** # \| Chosen Name \| Age \| Diagnosis \| Regular School \| ***# Pictures in total*** \| ***# movies*** \| ***# pictures selected for the interview***; 1 \| Batman \| 7y2m \| ADHD \| Yes \| ***58*** \| ***2*** \| ***7***; 2 \| Filouke \| 6y \| DCD \| Yes \| ***209*** \| ***1*** \| ***52***; 3 \| Beyblade \| 9y \| DCD & ASD \| No \| ***148*** \| ***2*** \| ***35***; 4 \| Loeizui \| 7y6m \| ASD & ADHD \| Yes \| ***457*** \| ***12*** \| ***61***; 5 \| Noem \| 6y6m \| ADHD & DCD & ASD \| Yes \| ***177*** \| ***0*** \| ***32***; 6 \| T-Rex \| 5y5m \| DCD \| Yes \| ***953*** \| ***0*** \| ***45***; 7 \| Miekie \| 7y7m \| DCD \| Yes \| ***248*** \| ***37*** \| ***27***; 8 \| Bent \| 7y4m \| ASD & ADHD \| Yes \| ***231*** \| ***2*** \| ***44***; 9 \| Nardas \| 6y8m \| ASD & DCD \| Yes \| ***451*** \| ***0*** \| ***84***; 10 \| Mortis \| 6y2m \| ASD \| Yes \| ***127*** \| ***0*** \| ***15***; 11 \| Ben \| 7y5m \| ASD & ADHD \| Yes \| ***79*** \| ***0*** \| ***7***; 12 \| Minnie \| 6y7m \| DCD \| Yes \| ***393*** \| ***0*** \| ***68***; 13 \| Rudy \| 7y2m \| DCD & ASD \| Yes \| ***221*** \| ***0*** \| ***37***; 14 \| Lientje \| 5y5m \| ASD \| Yes \| ***351*** \| ***8*** \| ***54***; 15 \| Thomas \| 6y5m \| ASD & ADHD \| No \| ***152*** \| ***0*** \| ***31***; 16 \| Zorro \| 7y \| ADHD \| Yes \| ***229*** \| ***4*** \| ***73*** In the results section, on line 332, we added the following sentences: ***In total, 3912 photographs and 68 short movies were made. It appeared that children loved the assignment as they took a lot of pictures. They enjoyed taking selfies with friends and families. The altered their pictures by adding funny things to their pictures such as harts or frames and such. Children also took photographs of a variety of activities including pets, nature, favorite games and such.* *Before conducting the second interview, we asked the children to make a selection of the most important pictures that would help them to guide the interview. A short summary of the reoccurring pictures can be seen in Appendix 3.*** In the limitation section, on line 698 we added: ***As well, we only focused on a time period of ‘one week’ rather than following these children during a longer period in time. More time in the field to gather longitudinal and observational data would capture participation across the different seasons or times of the year. As well, gathering data from other groups of participants such as those with differences in urban-rural, gender, ethnicity is still necessary.*** |
| Themes seem to be presented differently with some themes being larger than others, with some sub themes such as activity materials hardly consisting of a paragraph which is problematic as it does not present enough data to convince robustness. | We changed this in the document so that all themes are equally mentioned.  It is indeed correct that that themes such as activity materials hardly have a lot of sentences. This is because the children found it hard to put into words why they enjoyed these toys. It seemed that the children showed the importance of this theme by taking so many pictures of their toys. We hope that we made it more clear in the manuscript know. | In the results section, on line 352 we added:  While playing ***with other children***, ***they perceived*** ‘friendship’ and ‘companionship’ and felt ‘included’**. *When they played on their own in their home, the children enjoyed the sensory motor aspects of the toys. Hence, playing could be divided into two subthemes, more specifically friendship and features of activity materials.***  We added in the results section on line 490: ***When probed as to why the children chose these toys and activities, it was hard for them to put this into words. The children preferred to show the pictures and they took a lot of pictures of their toys. Examples are Lego, Playmobil, cars, trains and dinosaurs just to name the most common photographed toys.*** |
| Some themes appear to be unresolved-for example under the theme of learning there is some data about sports and participation that does not clearly relate to learning- more explanation of why it is analyzed and situated underneath this theme is needed. | Throughout the entire manuscript, more specifically in the methods, result and discussion section, we tried to ameliorate the manuscript so that it became more clear. We hope that this meets your expectations.  To specifically address this remark, we added extra information in the results section. | In the results section on line 416 we added: ***According to them, learning was defined as being better at something (on social, motor or cognitive skills).*** |
| Furthermore, the sub theme on environmental factors is unclear in its present form and needs more explanation. | **Regarding the subtheme environmental factors:**  As the main focus was to gather the perception of the children, we did not really dig deeper into the definitions of how environmental factors or contextual factors should be defined according to the children.  We only focused on how the children define what they perceived as facilitators or barriers. However, we agree with the remark made. It is indeed something that needs to be clarified in future research. Hence, we added this as a limitation to our study. | To meet this remark, we changed the visual presentation of the themes. We hope that this is more clear now.    In the results section on line 563, we added a different Figure 2    We added in the discussion section on line 581 the following: This research also revealed that participation did not just happen within a frame of contextual ***or environmental*** factors, but that those factors served, together with the parents and peers, as gatekeepers or mediators to the participation of children with DD in activities.  In the discussion section we added on line 591: This may suggest that little obstruction is perceived by the children thanks to the vast support of primarily their mothers ***and other contextual or environmental factors*.** |
| Overall the themes need to reflect multiple dimensions to be considered robust-to include the negative as well as positive sides for examples, or to include opposite opinions among the children's data as well as things they agreed with. | **Regarding the remark that “the themes need to reflect multiple dimensions to be considered robust to include the negative as the positive sides**… “ we agree. It is striking that children were only positive. We therefore mentioned this in the limitation section as this aspects needs more research to untangle it. | In the limitation section on line 683 we added: ***Another possible limitation is that , according to Docherty & Sandelowski (1999) children of all ages might withhold emotion-laden information. It seems that children tend to mask negative feelings or disappointment because they do not want to elicit a negative response from the interviewer or others who might hear what they say. To overcome this, we made sure to avoid the display of feelings such as surprise or horror at what the child was reporting. Children were also reassured that their personal stories would not be revealed to the people (especially parents) they are trying to protect them from these negative feelings. It might have been that by not including parents in the room where the interview took place, that the children would have reported more negative feelings than they currently did.*** |
| Finally the discussion would need more focused work once the findings are more clearly described. | We worked on this remark and we hope that the discussion is more in line with what you suggest. | We made changes throughout the entire manuscript to ameliorate the clarity of the manuscript. We hope that this meets your expectations. See below |
| There are a few assumptions being made in the discussion  For example, it is important to note that children with DD have been involved in other studies similarly about their engagement in activities and their experiences, so the authors need to say that this is the first study to their knowledge, (line 514). | It is indeed an assumption. We changed it in the manuscript. | We added the following in the discussion section on line 587: While some of the findings of this study are not surprising given recent systematic review of the literature, ***to our knowledge***, this is the first study to specifically ask young children with DD about their own subjective experience of participation. |
| Also, an assumption is made that mothers aren't able to enable participation as the child gets older (line 533), this needs to be reconsidered. | It is indeed an assumption. We changed it in the manuscript. | We also deleted in the discussion section on line 646: Similar results are in line with the findings of Jasmin, Tetreault, Lariviere and Joly (Jasmin 2018), proving that ***~~indeed~~***, both perceptions ***between children and parents*** might differ in that aspect.  In the discussion section on line 650 we changed the sentence by adding and deleting words: It also could be that, as children grow older, the gap between the child’s capacity and requirements to be able to participate enlargers***.*** ***It could be that*** mothers ***are afraid to be less able*** to enable their child’s participation resulting in a decreased sense of participation in older children with DD. This however, needs further longitudinal research to untangle this idea. |
| Consent- as all studies require consent, this is not the most useful or insightful limitation to make in the final limitation section. Reconsider including other limitations such as more time in the field to gather longitudinal data which would therefore capture participation across the different seasons or times of the year, or to gather data from other groups such as those with differences in urban-rural, gender, ethnicity. | This is a very good point. We tried to adjust this into the manuscript. We hope it is more clear now. | We added the following in the limitation section on line 683: (see also above) ***Another possible limitation is that, according to Docherty & Sandelowski (1999) children of all ages might withhold emotion-laden information. It seems that children tend to mask negative feelings or disappointment because they do not want to elicit a negative response from the interviewer or others who might hear what they say. To overcome this, we made sure to avoid the display of feelings such as surprise or horror at what the child was reporting. Children were also reassured that their personal stories would not be revealed to the people (especially parents) they are trying to protect them from these negative feelings. It might have been that by not including parents in the room where the interview took place, that the children would have reported more negative feelings than they currently did.***  In the limitation section on line 698 we added the following: ***As well, we only focused on a time period of ‘one week’ rather than following these children during a longer period in time.. More time in the field to gather longitudinal and observational data would capture participation across the different seasons or times of the year. As well, gathering data from other groups of participants such as those with differences in urban-rural, gender, ethnicity is still necessary.*** |
| Although the paper is well referenced, consider looking at more of the literature on happiness as an indicator of well-being in young children to inform a more nuanced data analysis outcome and discussion. This aspect appeared to be evident in your data but not considered from this perspective. | Thank you for the useful tip to consider looking at the literature on happiness as an indicator of well-being. We added this in our manuscript. | In the discussion section on line 622 we added the following  ***Fourth, it seems worthwhile to further investigate whether the construct of happiness and participation could be related. The term happiness is used in the context of mental or emotional states, including positive or pleasant emotions ranging from contentment to intense joy.***  ***According to Antaramian and colleagues (2010) traditional mental health models focus on psychological problems and distress. The recent emergence of positive psychology has placed increased emphasis on the positive factors, such as life satisfaction and happiness***  ***(Antaramian et al. 2010; Seligman and Csikszentmihalyi 2000). Given the importance of positive indicators of emotional well-being, particularly acknowledging that high happiness contributes to determining remission of psychological symptoms, the relationship between happiness and participation should be further investigated.*** ***Given the fact that young children with DD have often adaptive-behaviour deficits, it seems worthwhile to further investigate the clinical use of indices of happiness offers. Quite simply, the absence of indices of happiness or presence of indices of unhappiness can cause occasional changes in programming. By observing affective behaviour, school, leisure, and therapeutic activities, can be added to or subtracted from***  ***the child’s support plan to ensure more or better participation possibilities.*** |
| **From the second Reviewer** |  |  |
| it was a pleasure to learn from your work. We would suggest following elements to bring the article on 'the next level'. | Thank you for this remark. It is motivating to read that we are able to improve this manuscript. |  |
| on page 4, line 79 you were bringing research into ' ' : I would suggest to skip the ' ' as it can be seen as an act of non-respect to your peers | This is a correct remark. We deleted the “ “ in the manuscript. Sorry for this. | In the introduction section, on line 72 we deleted the ‘ ‘Their narrative systematic review concluded that the participation phenomenon is essentially dichotomous- requiring children to ***~~“~~***attend***~~”~~*** (be present) and to be ***~~“~~***involved***”*** (engage, experience and so forth) (Maciver 2019). |
| at page 6 you conclude that the opinion of children is not yet studied and you are calling this ...a surprise...: are there any reasons why this voice is not put forward? And is the idea of children having 'their own voice' not a bit naive--> I think that more can be said to make sure the surprise gets context | Thank you for this remark. We hope that by adding and elaborating on this matter, we gave the ‘surprise’ more context. | In the introduction section on line 80 we added the following sentences: ***This could be due to the fact that only recently, the World Health Organisation (WHO) developed the International Classification of Functioning-Child Youth (ICF-CY). The development of ICF-CY originates from the need to describe the functioning of children and youth in correspondence with International Classification of Functioning (ICF). The ICF-CY offers especially more extended points of departure and greater detail for a better description of the growth and development of children and youth.*** ***This might have resulted in the fact that*** up till now, the voices of children with disabilities were absent in disability studies (Whyte 2005). |
| photo voice projects are more and more using personal phones as a tool instead of digital camera's: did you think about this option? | As we were interested in collecting data from young children, we opted for the use of digital camera’s as young children of 5 or 6 years old rarely have their own smartphone. For older children, that might be a good suggestions as the quality of the pictures with a digital camera that we used is not the best. Thank you for pointing this out. We will use this feedback for future research. | We added in the manuscript, in the methods section on line 169 the following: ***As most young children in Belgium are not yet owners of a smartphone, we chose to collect data with a robust digital camera that is child friendly.*** |
| how did you prepare the children about the ethical standards necessary to work with photo voice?? | During the first interview, we explained the children the use of the camera. While doing so, we also mentioned that they needed to ask permission first of the person that they wanted to take a picture from. As well, we made information letters for the school and the sports clubs or other recreational settings so that these settings were informed and were able to consent voluntary. | We added this information into the methods section on line 225: ***During the first interview, we explained the children the use of the camera. While doing so, we also mentioned that they needed to ask permission first of the person that they wanted to take a picture from. As well, we made information letters for the school and the sports clubs or other recreational settings so that these settings were informed and were able to consent voluntary.*** |
| . if children felt the comic strip style of report was a bit childish , could they get an alternative version? | We did not perceive any comment from the parents nor the children regarding the comic strip as being ‘childish’. On the contrary, the children enjoyed the fact that the research paid attention to them, that he read the comic strip and played a game or made a drawing together with the child. Parents perceived it as positive as they used the comic strip during that first week.  However, it is indeed a good suggestion to have a back-up plan in case the comic strip is perceived childish. We for sure will take this remark into account when conducting future research, especially with older children. | We did not made any changes into the manuscript for this remark. |
| What was the rationale to use Thematic Analysis as analytic tool? | This is a correct remark. We did not really specify the rationale. We therefore added it in the manuscript. | In the methods section on line 180 we added the following: ***We chose for a thematic analysis as it is still the most useful in capturing the complexities of meaning within a textual data set (Creswell, 2009).*** |
| On page 11 you are offering a good overview about the members of your research team; we did not get information about 'the position' of this team visà vis the children involved in your study | That is correct. We made changes to ameliorate the manuscript. | In the methods section on line 273 we added:  ***The research team had no connection with the children nor their parents as clinicians nor family ties.*** |
| Is it necessary/helpful to bring in the diagnosis of the children linked to their quotes (this sound very medical model) | This is a very good remark. It indeed reflects a more medical view and it is not necessary for the reader. We therefore deleted this in the entire manuscript. | We made changes in the following lines in the results section:  On line 357: Loeizui, a 7,5 year old boy ***~~with ADHD and ASD~~***, told us, after we asked him why person X was his friend, that he liked his friend *“because he laughs with me and because he wants to play with me and we tickle each other all the time”.*  On line 375: Nardas, a boy of 6 years old ***~~with ASD and DCD~~***, simply answered: *“Because we know each other so long. We don’t know each other* *well. But because he is my best friend, I know. We are since day care together, and we are now also in the same class.”*  On line 409: Noem, a 6 year old boy ***~~with ADHD, DCD and ASD~~*** quoted: *“I like to go to school because I can learn. It goes easy. I made a test and I was the best of the class.*  On line 422: Same for Bent, 7 years old ***~~with ASD and DCD~~*,** who loves going to the boys scouts. “*I like going to the scouts, I have to make a square”* (he means formation at the start of the activity).  On line 458: Minnie, a 6 year old girl ***~~with DCD~~*** was vividly talking about her trip with her dad.  On line 475: Filouke, a 6year old boy ***~~with DCD~~***, said *“I love getting up in the morning as the first. Then I can feed the fish and the cat”.*  On line 488: Rudy, 7 years of age ***~~and diagnosed with ASD and DCD~~*** and going to a school specialized in children with autism, explained that he wanted to go back to his previous school as at that school he wasn’t forced to learn to play with other children.  On line 516: When asked what Filouke, 6 year ***~~and diagnosed with DCD,~~*** wanted to change regarding his participation, he articulated this nicely by telling that he did not want to fall anymore. |
| Was it an option to bring in certain pictures in the text to make your report more vivid? | The quality of the taken pictures are not good for printing purposes. The children enjoyed taking pictures but were not able nor interested in making sharp pictures. Therefore, we decided not to include pictures in the manuscript. We did however make a summary of the most common themes in the pictures in Appendix 2.  From this perspective, we hope it will be more clear to the reader what it was all about. |  |
| Technical: you named figure 2, figure 1 | We have made the changes. | The following change has been made:  *Figure* ***2*** *about here* |
| Why do you skip the 'survival strategies of the kids' (walking away,...) in your figure 2? Now it sticks To the successful participation. We all know that 'failures' can help to learn, and that a lot of the kids know failures very well. | Thank you for pointing this out. It is indeed a good suggestion to include this into the figure. We made the suggested changes and hope that is more complete now. | On the results section on line 563 we changed the content of Figure 2.   |
| I think that in your conclusions the Self Determination Theory gets too much attention. I can agree with the fact that researchers are looking for frameworks that help to bring elements together, but... now you are ignoring great literature about 'recognition' (Judith Butler e.g.) and there is an enormous amount of interesting literature about 'belonging' | Thank you for pointing this interesting literature. I was indeed not aware of this theory. I read it with interest and made changes accordingly. I hope that my understanding of the theory and the implication to this study, is made correctly. | In the abstract on line 42 we made the following changes: These themes fit well within earlier and current research on the subject of participation ***~~and within the Self-Determination Theory.~~***  In the discussion section, on line 594 we added  ***This finding is also in line with others theories such as 1) the Self-Determination Theory (SDT)*** *(Ryan & Deci, 2017)****, 2) the theory regarding Belonging, 3) theory on Recognition and on 4) Happiness that are not commonly used nor known in the rehabilitation guiding theories and guiding frameworks. It would be interesting to conduct further research on the use of these theories to better guide clinical pediatric rehabilitation***.  ***First, the theory regarding the SDT, more specifically*** the feeling of autonomy, belonging and competence seems important ***to include when developing interventions as health care clinicians. The findings of this study indicate that the benefits of SDT are*** not only ***interesting*** for the engagement and well-being of regular students. This study seems to indicate the value ***of including the principles of SDT*** for children with DD as well.  ***Second, a same argument can be made regarding the theory of Belonging. According to Wright (2014) “belonging” has been described as a puzzling term. Feeling a sense of belonging (or not), being morally, socially or legally recognized as belonging (or not), could have the power to change lives. As the literature on belonging is currently very diverse, it might be interesting to further investigate how this construct relates to participation and young children with DD.***  ***Third, according to Leopold (2018), “recognition” is a key concept in contemporary social and political thought. The fundamental insight associated with this concept is that we cannot really be who we are or who we want to be if others do not treat us in certain ways. Leopold (2018) argues that we depend on others or, more specifically on the recognition by others, in order to be able to actualize our identities. She argues that if we do not find recognition in our personal interactions or in the wider society, we are peculiarly constrained in our being.***  In the discussion section on line 637 we added: ***To conclude, looking further into detail in how these theories can enhance our understanding seems important*** as it advances thinking by identifying specific factors (i.e. playing, learning and family gatherings) that decision-makers and interventionists may consider to help children ***to feel that they belong, are recognized, happy recognized and therefore be better equipped to*** participate.  In the discussion section on line 643 we deleted the following:  ***This finding is in line with the self-determination theory (Ryan & Deci, 2017). The feeling of autonomy, belonging and competence seems important not only for the engagement and well-being of regular students: this study seems to indicate the value for children with DD as well. This analysis is of key issues as it advances thinking by identifying specific factors (i.e. playing, learning and family gatherings) that decision-makers and interventionists may consider to help children to participate.***  In the conclusion section on line 673 we deleted the following: *“These themes fit well within earlier and current research on the subject of participation and within the self-determination theory.* |
| On page 26 you are observing the fact that service providers are not in the stories and you are bringing this back to the culture of Flemish support giving services. I can't follow this linear way of thinking. I think that younger children always stick to the concrete elements of a procedure (most interviews were done at home with parents) - a lot of children are living in middle class families (with a lot of time and car energy). I would suggest to look in that direction. | I can understand this remark. As the focus of our study was not specifically on this issue, we decided to delete this from the discussion section. | In the discussion On line 667 we deleted the following:  ***Another novel insight that came from this study was that the children with DD did not mention the impact of providers, even though almost all children received health care. This was an unexpected finding since currently healthcare providers claim to be more working focusing on participation, something children did not yet experience. It could be that, while internationally health care practice is moving towards the adoption of a system or ecological view, the field in Belgium still operates predominantly from a unidirectional perspective where “something” is provided to “fix” the person with a disability (Fauconnier, Dickinson et al. 2009) rather than operating from more contemporary view of participation outcomes.*** |
